# EGR1‐mediated linc01503 promotes cell cycle progression and tumorigenesis in gastric cancer

**DOI:** 10.1111/cpr.12922

**Published:** 2020-11-03

**Authors:** Zhonghua Ma, Xiangyu Gao, You Shuai, Xiaolong Wu, Yan Yan, Xiaofang Xing, Jiafu Ji

**Affiliations:** ^1^ Key Laboratory of Carcinogenesis and Translational Research (Ministry of Education) Division of Gastrointestinal Cancer Translational Research Laboratory Peking University Cancer Hospital and Institute Beijing China; ^2^ Department of Medical Oncology Jiangsu Cancer Hospital Jiangsu Institute of Cancer Research The Affiliated Cancer Hospital of Nanjing Medical University Nanjing China; ^3^ Department of Endoscopy Center Key Laboratory of Carcinogenesis and Translational Research (Ministry of Education) Peking University Cancer Hospital and Institute Beijing China

**Keywords:** cell cycle, EGR1, gastric cancer, Linc01503, lncRNA, tumorigenesis

## Abstract

**Objectives:**

Long non‐coding RNAs (lncRNAs) are key mediators in various malignancies. Linc01503 was previously elucidated to promote gastric cancer (GC) cell invasion. However, the upstream mechanism of linc01503 and its involvement in GC cell cycle, apoptosis and tumorigenesis still remain unclear.

**Materials and Methods:**

Bioinformatics analysis and quantitative reverse transcription polymerase chain reaction (qRT‐PCR) assays were implicated to detect linc01503 level in GC. The role of linc01503 was detected by in vitro functional assays and in vivo xenograft tumour models. The association between linc01503 and its upstream effector was identified by chromatin immunoprecipitation (ChIP) assays. The mechanistic model of linc01503 was clarified using subcellular separation, fluorescence in situ hybridization, RNA‐sequencing, RNA immunoprecipitation (RIP) and ChIP assays.

**Results:**

Linc01503 was remarkably elevated in GC and tightly linked with the overall survival of patients with GC. The key transcription factor early growth response protein 1 (EGR1) critically activated the transcription of linc01503. Functionally, linc01503 knockdown resulted in the activation of apoptosis and G1/G0 phase arrest in GC. Mechanistically, linc01503 interacted with histone modification enzyme enhancer of zeste 2 (EZH2) and lysine (K)‐specific demethylase 1A (LSD1), thereby mediating the transcriptional silencing of dual‐specificity phosphatase 5 (DUSP5) and cyclin‐dependent kinase inhibitor 1A (CDKN1A) in GC.

**Conclusions:**

EGR1‐activated linc01503 could epigenetically silence DUSP5/CDKN1A expression to mediate cell cycle progression and tumorigenesis, implicating it as a prospective target for GC therapeutics.

## Graphical abstract

The EGR1‐activated lncRNA linc01503 mediated gastric carcinogenesis through the axis of EZH2/LSD1/DUSP5/CDKN1A. The mechanistic model of linc01503 is a further prerequisite for progress in translational exploitation of lncRNAs for treatment of patients with GC.

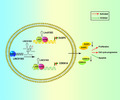

## 1 INTRODUCTION

Gastric cancer (GC) is the third leading cause of cancer‐related death worldwide and poses a great threat to the public health.^1^ Based on the global cancer statistics in 2018, about 1.03 million GC cases and 0.78 million deaths were estimated in that year.^1^ The high mortality from GC is mainly due to its advanced‐stage diagnosis, and thus, early detection and treatment are extremely important.^2^ Therefore, there is an urgent need to enrich the knowledge associated with GC biology and to identify effective tumour markers and therapeutic targets for patients with GC.

Long non‐coding RNAs (lncRNAs) are a subset of non‐coding RNAs (ncRNAs) longer than 200 nucleotides, with little or no protein‐coding potential.^3^ Importantly, lncRNAs have been considered as crucial regulators in multiple cancers and participate in a wide range of biological activities.^3^ Emerging evidence has demonstrated that lncRNAs could induce gene regulation through the interplay with various biomolecules or recruitment of chromatin‐modifying complexes.^4^, ^5^ Moreover, the regulatory association between lncRNAs and transcription factors also enrich the knowledge of the upstream regulatory mechanisms of lncRNAs in various types of cancers.^6^, ^7^ For instance, lncRNA HCCL5 was transcriptionally driven by zinc finger E‐box–binding homeobox 1 (ZEB1) in hepatocellular carcinoma (HCC).^8^ Our previous research elucidated that C8orf76 specifically targeted lncRNA DUSP5P1 for its transcriptional induction, thereby activating the MAPK/ERK signalling pathway to promote gastric tumorigenesis.^9^ Thus, deep identification of mechanisms associated with GC progression is of great significance to develop future diagnostic and therapeutic approach.

Linc01503 is a novel lncRNA located in human chromosome 19 and potentially correlated with human cancer development.^10^, ^11^, ^12^ Linc01503 was firstly uncovered to serve as an oncogene in aggressive squamous cell carcinoma (SCC).^12^ Another research directed by Lu et al uncovered that linc01503 could sponge miR‐4492 to mediate forkhead box K1 (FOXK1) expression, leading to the malignant progress of colorectal cancer (CRC).^13^ Moreover, linc01503 was characterized to enhance the malignant behaviours in cholangiocarcinoma, glioma, cervical cancer and hepatocellular carcinoma.^11^, ^14^, ^15^, ^16^ Strikingly, He et al reported that androgen receptor‐activated linc01503 promoted nasopharyngeal carcinoma progression through the splicing factor proline‐ and glutamine‐rich (SFPQ)/fos‐like 1 (FOSL1) axis.^17^ Recently, Ding et al detected the alteration of GC cell numbers upon linc01503 dysregulation and proposed that linc01503 could promote GC cell invasion through inducing wnt signalling pathway.^10^ This research by Ding et al highlighted the critical role of linc01503 in GC development and progression.^10^ However, the detailed impact of linc01503 on GC cell proliferation, cell cycle progression, in vivo tumorigenesis and the mechanistic model of linc01503 in GC still require further identification.

Here, we provided the first evidence of the upstream mechanism of linc01503 in GC and further enriched the functional role and mechanistic models of linc01503 in GC. Firstly, bioinformatics analysis predicted a significantly altered expression of linc01503 between GC tissues and non‐tumour tissues. This perspective was subsequently verified by quantitative reverse transcription polymerase chain reaction (qRT‐PCR) assays. Also, it was notable that increased level of linc01503 tightly associated with worse overall survival (OS) of GC patients. Intriguingly, the key transcription factor early growth response protein 1 (EGR1) critically activated the transcription of linc01503 in GC. Functional assays indicated that silencing of linc01503 markedly mediated GC cell proliferation and apoptosis both in vitro and in vivo, and induced the alteration of GC cell cycle regulation. Next, RNA‐sequencing (RNA‐seq) combined with gene set enrichment analysis (GSEA) showed that linc01503 knockdown effectively affected genes associated with cell cycle and apoptosis, potentially contributing to the promotion of GC proliferation. Mechanistically, the driving forces of linc01503 were regulated, at least partially, by interacting with enhancer of zeste 2 (EZH2) and lysine (K)‐specific demethylase 1A (LSD1). Importantly, linc01503 was elucidated to epigenetically suppress dual‐specificity phosphatase 5 (DUSP5) and cyclin‐dependent kinase inhibitor 1A (CDKN1A) expression through binding with EZH2 and LSD1 in GC. In summary, our study largely enriched the functional role and novel mechanism of linc01503 in GC and the axis of EGR1/linc01503/DUSP5/CDKN1A may be an attractive target for GC therapeutics.

## 2 MATERIALS AND METHODS

### 2.1 Clinical samples and cell lines

GC specimens were collected from Peking University Cancer Hospital with informed consent policy. The research was approved by the Ethics Committee of Peking University Cancer Hospital and conducted under the guidance of the Declaration of Helsinki. The detailed information of cell lines is summarized in Appendix S1.

### 2.2 RT‐qPCR assays

Total RNA was extracted in TRIzol reagent (Invitrogen) based on the descriptions of the product. The detailed information is summarized in Appendix S1. The used primers are listed in Table S1.

### 2.3 Cell transfection

Transfections were performed using Lipofectamine 3000 (Invitrogen) based on the instructions of the manufacturer. The detailed information is summarized in Appendix S1. The information for siRNAs oligonucleotides is listed in Table S1.

### 2.4 Animal models

Animal experiments were approved by the Animal Ethics Committee at Peking University Cancer Hospital. The detailed information is summarized in Appendix S1.

### 2.5 RNA immunoprecipitation (RIP)

The interactions between linc01503 and potential binding proteins were detected by RIP assays using the EZ‐Magna RNA Immunoprecipitation (RIP) Kit (Millipore) under the instructions of the manufacturer. The details are summarized in Appendix S1.

### 2.6 Chromatin immunoprecipitation assays (ChIP)

According to the protocol of the manufacturer, ChIP assays were performed with a MagnaChIP Kit (Millipore). Detailed information is summarized in Appendix S1. The details of used antibodies are listed in Table S1.

### 2.7 Subcellular separation and fluorescence in situ hybridization (FISH) assays

Subcellular separation assays were performed with a PARIS Kit (Life Technologies) in accordance with the protocol of the manufacturer. FISH assays were performed with a Ribo™ Fluorescent In Situ Hybridization Kit (RiboBio) according to the manufacturer's instructions. The detailed information is summarized in Appendix S1.

### 2.8 Statistical analysis

The differences in groups were assessed with Student's *t* test, Wilcoxon's test and *χ* ^2^ test, as appropriate. The association between EGR1 and linc01503 was evaluated using Spearman's correlation analysis. The data were manifested as mean ± standard deviation (SD) and analysed using SPSS 21.0 software (IBM). All tests were two‐sided, and *P* < .05 was considered to indicate a significant difference.

## 3 RESULTS

### 3.1 lncRNA linc01503 was highly expressed in GC and tightly related to the survival outcome of patients with GC

**FIGURE 1:**
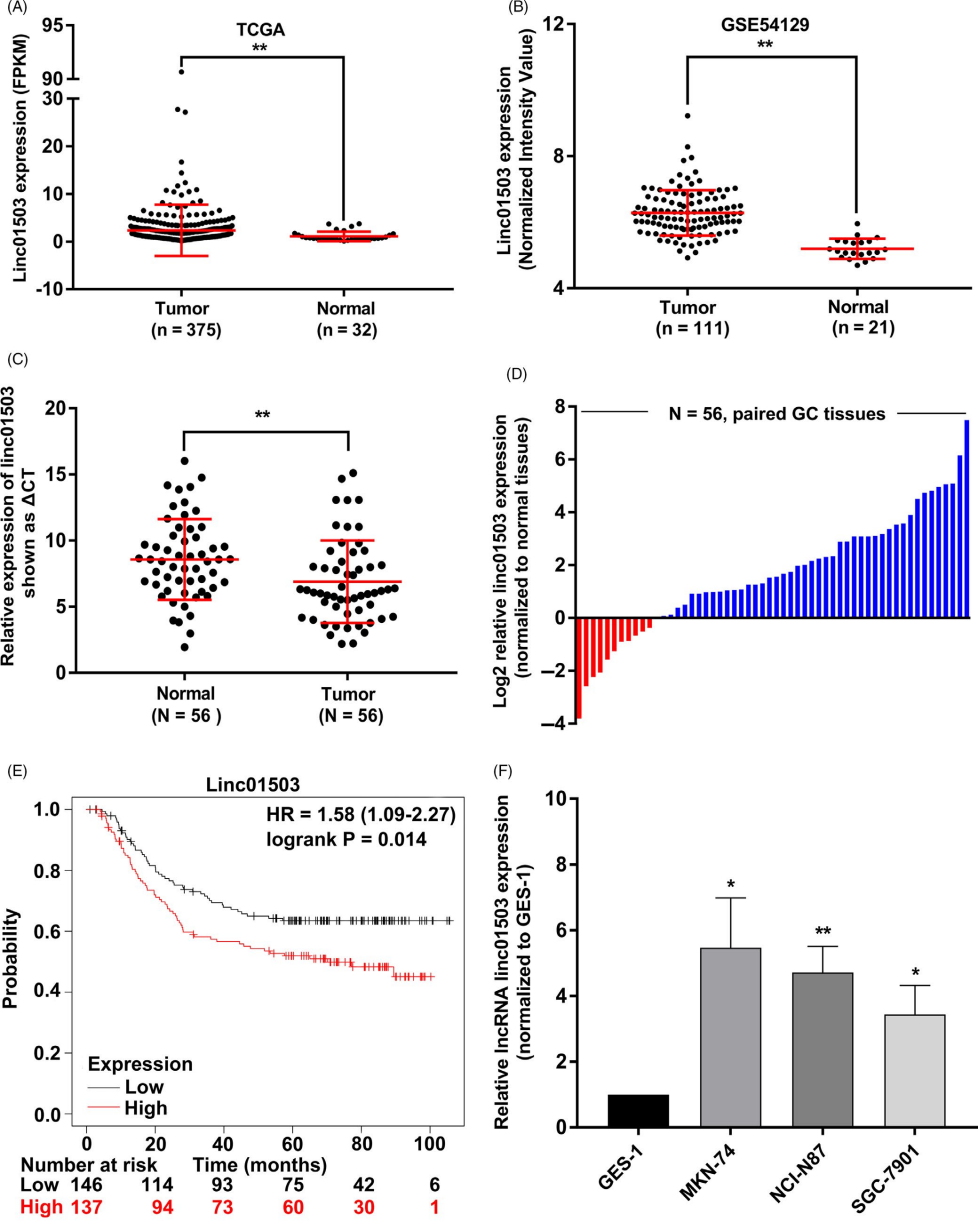
lncRNA linc01503 was markedly elevated in GC and tightly linked to the survival outcome of patients with GC. A and B, The expression profile of linc01503 in GC tissues and normal specimens was detected through analysing the data from TCGA and GEO (GSE54129) data sets. C and D, Linc01503 level was verified in 56 paired GC tissue specimens using qRT‐PCR assays. E, The relationship between linc01503 level and overall survival of GC patients (n = 283) was identified using Kaplan‐Meier plotter. F, Linc01503 was remarkably increased in GC cell lines. *P < .05, **P < .01

We firstly analysed the publicly available data from The Cancer Genome Atlas (TCGA) and Gene Expression Omnibus (GEO) DataSets and aimed to find candidate lncRNAs potentially correlated with GC progression. The bioinformatics analysis showed that linc01503 displayed remarkable upregulation in GC tissues compared with non‐tumour tissue samples (Figure 1A, B). To verify the expression pattern of linc01503 in GC, we performed qRT‐PCR assays in a cohort of 55 paired GC tissues and adjacent non‐tumour tissues. Consistently, linc01503 was dramatically increased in GC tissues relative to non‐tumour specimens (Figure 1C, D). Further, we evaluated the expression level of linc01503 in GC cells lines and found that linc01503 level displayed significant upregulation in GC cells compared with GES‐1 cells, highlighting the critical involvement of linc01503 in GC progression (Figure 1F).

To further clarify the relationship between linc01503 level and survival outcome of patients with GC, the Kaplan‐Meier plotter (KmPlot), which has been used in various cancers, was implicated in this research (http://kmplot.com/analysis).^18^, ^19^, ^20^, ^21^, ^22^A total of 283 GC patients were divided into linc01503 high group (n = 146, fold change ≥ median value) and linc01503 low group (n = 137, fold change ≤ median value). As shown in Figure 1E, KmPlot analysis indicated that higher linc01503 level tightly associated with worse OS, using publicly obtained data from 283 GC patients (Figure 1E). Overall, the elevation of linc01503 in GC may be of great significance for GC therapeutics.

### 3.2 EGR1 critically activated the transcription of linc01503 in GC

**FIGURE 2:**
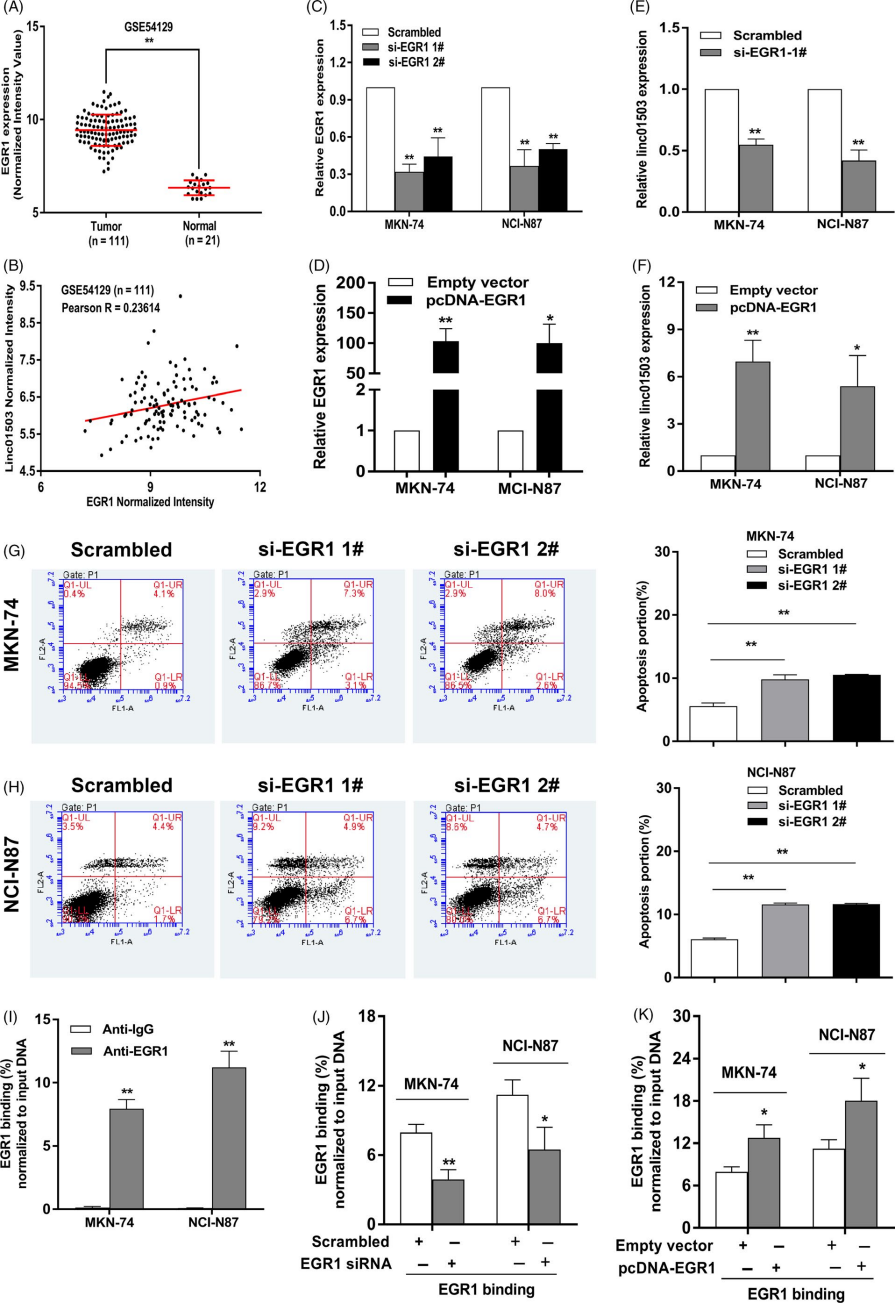
The key transcription factor EGR1 critically activated the transcription of linc01503 in GC. A, The expression pattern of EGR1 was detected in the data obtained from GEO (GSE54129) database. B, The correlation between the expression level of linc01503 and EGR1 in GC tissue samples was evaluated based on the publicly available data from GEO (GSE54129) database. C and D, The expression level of EGR1 was detected in GC cells transfected with EGR1 siRNAs or pcDNA‐EGR1. E and F, The impact of the altered expression level of EGR1 on the regulation of linc01503 expression in GC. G and H, The impact of EGR1 silencing on GC cell apoptosis. I, The ChIP‐qPCR assays revealed the direct binding of EGR1 to endogenous linc01503 promoter regions in GC cells. J and K, The ChIP‐qPCR assays revealed the enrichment of EGR1 on linc01503 promoter in GC cells transfected with EGR1 siRNAs or pcDNA‐EGR1 in GC cells. *P < .05, **P < .01

Nowadays, massive studies have highlighted the dysregulation of lncRNAs in multiple malignancies.^10^ Nevertheless, the upstream mechanistic models of lncRNAs still require much attention. Here, we attempted to determine the regulators involved in linc01503 overexpression, thereby, at least partially, uncovering the upstream mechanism of linc01503 in GC. Notably, evidence has demonstrated that some transcription factors (TFs) could critically induce the transcription of some lncRNAs.^7^, ^23^ Here, we explored the publicly available data from GEO database and aimed to filter the key TFs potentially associated with linc01503 enrichment in GC. As shown in Figure 2A, the analysis of GSE54129 database elucidated the obvious increase of the transcription factor EGR1 in GC tissues (Figure 2A). Importantly, a positive association between linc01503 and EGR1 was highlighted in GC tissues, suggesting the potential regulatory correlation between EGR1 and linc01503 in GC (Figure 2B).

To verify the EGR1‐involved upstream regulatory mechanism of linc01503, we firstly implicated siRNAs against EGR1 or pcDNA‐EGR1 to reach EGR1 knockdown or overexpression in GC cells (Figure 2C, D). Then, qRT‐PCR assays were implicated to clarify linc01503 level in MKN‐74 and NCI‐N87 cells with EGR1 knockdown or overexpression. It was observed that silencing of EGR1 effectively impaired the expression level of linc01503 in GC (Figure 2E). By contrast, Overexpressed EGR1 impacted the opposite influence (Figure 2F). Meanwhile, it was worth noting that silencing of EGR1 could markedly activate the apoptotic activities in both MKN‐74 and NCI‐N87 cells (Figure 2G, H). The elevation of EGR1 could significantly inhibit the apoptotic activities in both MKN‐74 and NCI‐N87 cells (Figure S1A, B). Furthermore, we conducted ChIP assays and elucidated that EGR1 directly interacted with the EGR1 binding sites within the linc01503 promoter in GC cells (Figure 2I). Subsequently, we detected that the reduction or elevation of EGR1 could obviously decrease or increase EGR1 enrichment on the linc01503 promoter, respectively (Figure 2J, K), leading to linc01503 reduction or elevation in GC cells, respectively (Figure 2E, F). Collectively, the key transcription factor EGR1 was involved in the activation of linc01503, contributing to the increased level of linc01503 in GC.

### 3.3 Linc01503 regulated GC cell proliferation both in vitro and in vivo

**FIGURE 3:**
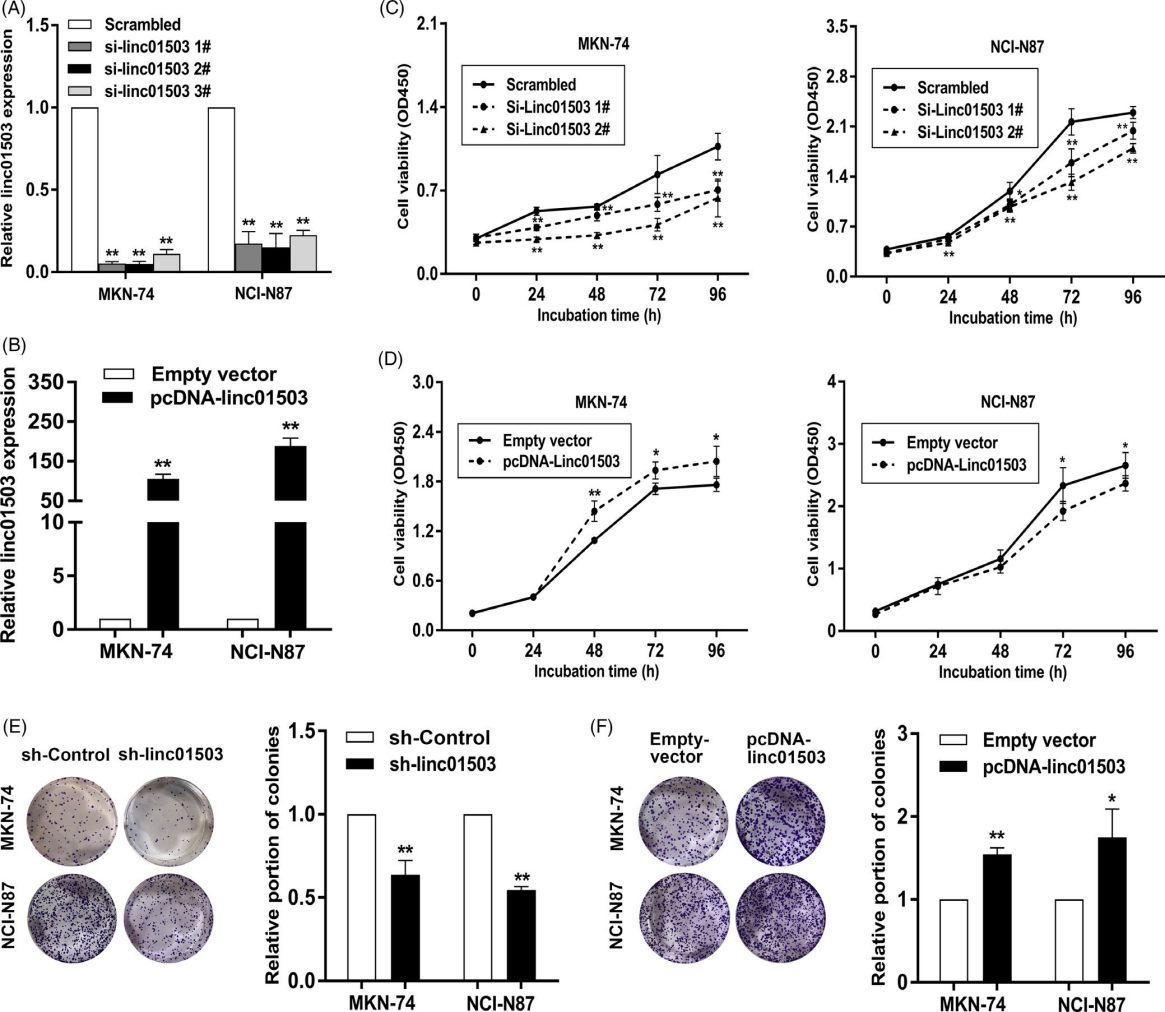
lncRNA linc01503 promoted GC cell proliferation. A and B, The efficacy of linc01503 knockdown or overexpression in GC cells was detected using qRT‐PCR assays. C and D, The impact of linc01503 reduction or elevation on GC cell viability. E and F, The impact of linc01503 reduction or elevation on the colony‐formation abilities of GC cells. *P < .05, **P < .01

To identify the biological role of linc01503 in GC, the effective knockdown or overexpression of linc01503 was made to perform loss‐of‐function and gain‐of‐function assays in GC cells (Figure 3A, B). Cell Counting Kit 8 (CCK8) analysis demonstrated that the depletion of linc01503 remarkably inhibited the proliferative capacities of GC cells (Figure 3C). This perspective was further verified by colony‐formation assays (Figure 3E). By contrast, overexpression of linc01503 played a promoting role in GC cell proliferation (Figure 3D, F).

**FIGURE 4:**
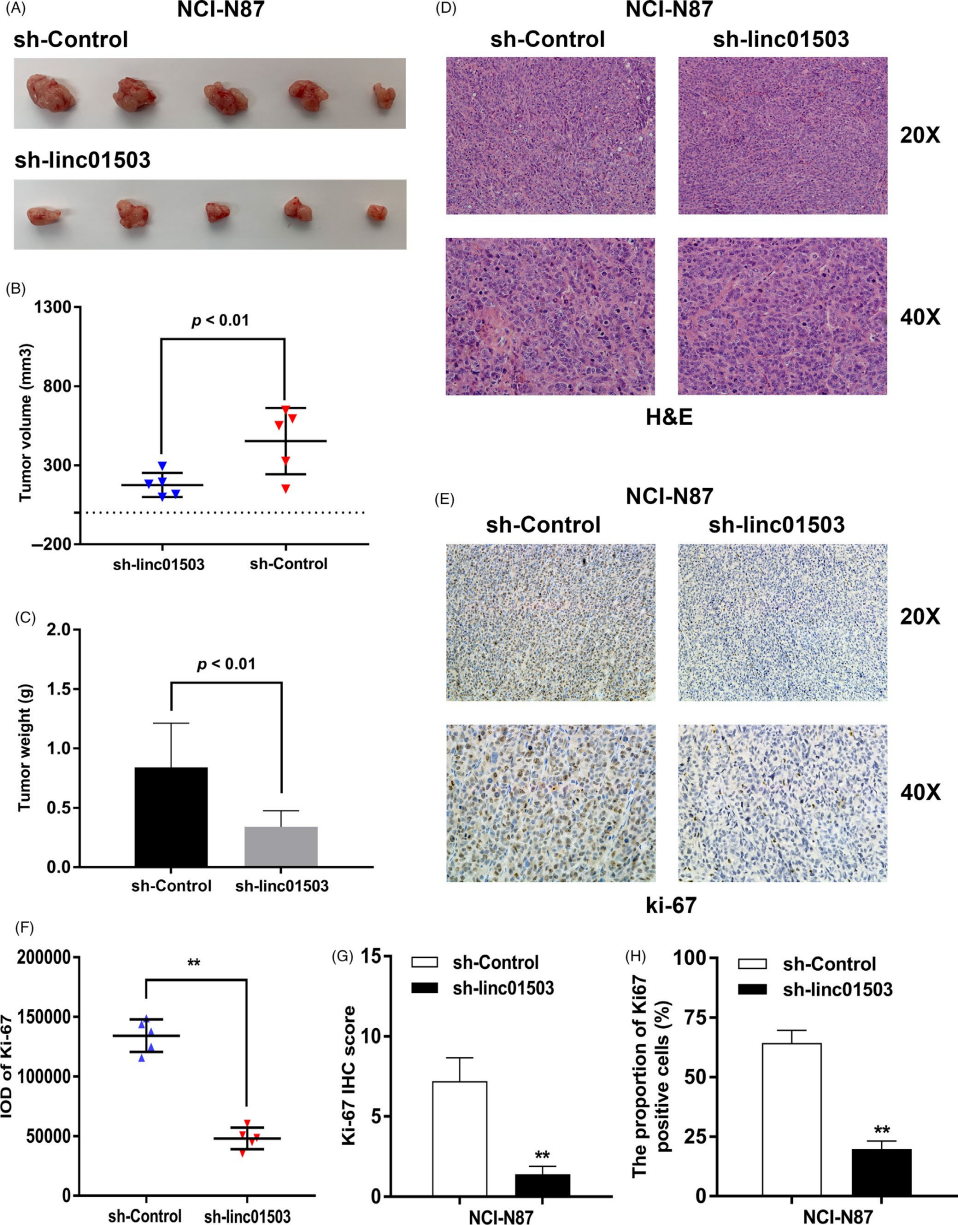
The impact of linc01503 knockdown on in vivo tumorigenic capacity of NCI‐N87 cells. A, The dissected tumours bearing from linc01503‐stable‐knockdown NCI‐N87 cells and control cells. B and C, The volume and weight of tumours obtained from linc01503‐stable‐knockdown NCI‐N87 cells and control cells. D and E, H&E and Ki‐67 staining of the tumours isolated from mice. 20× images and 40× images were shown. F, The IOD value of ki‐67 in tumours from sh‐control group and sh‐linc01503 group. G and H, Quantification of ki‐67 IHC score and proportion of ki‐67‐positive cells. The ki‐67 IHC score was calculated by multiplying the number corresponding to different ki‐67 staining intensity and staining regions. *P < .05, **P < .01

To further identify the impact of linc01503 on GC tumorigenesis in vivo, linc01503‐stable‐knockdown NCI‐N87 cells or control cells were subcutaneously injected into nude mice. Consistent with the in vitro assays, the depletion of linc01503 obviously inhibited the tumorigenicity in vivo (Figure 4A). It was shown that both the volume and the weight of tumours obtained from linc01503‐stable‐knockdown group were obviously reduced compared with the volume and weight of tumours derived from control group (Figure 4B, C). Furthermore, tumours formed from linc01503‐stable‐knockdown group displayed lower ki‐67 level than those from control group (Figure 4D, E). Specifically, quantitative statistical analysis was conducted to assess the ki‐67 level in tumours obtained from sh‐Control group and sh‐linc01503 group. As shown in Figure 4F, the integrated optical density (IOD) of ki‐67 levels in sh‐Control group were obviously elevated than those in sh‐linc01503 group (Figure 4F). Meanwhile, the ki‐67 IHC score and ki‐67‐positive percentages in sh‐Control group were significantly higher than those in sh‐linc01503 group (Figure 4G, H).

Then, we further detected the impact of linc01503 knockdown on the tumorigenic capacity of MKN‐74 cells in vivo. Similarly, the growth of tumours obtained form linc01503‐stable‐knockdown MKN‐74 cells was markedly inhibited compared with the growth of tumours from control groups (Figure S2A), together with the significantly impaired tumour volumes and weights (Figure S2B, C). Additionally, tumours acquired from linc01503‐stable‐knockdown MKN‐74 cells revealed lower ki‐67 levels than tumours from the control group (Figure S2D, E). In detail, the IOD of ki‐67, ki‐67 IHC score and proportion of ki‐67‐positive cells in tumours formed from linc01503‐stable‐knockdown MKN‐74 cells were markedly reduced than those in tumours from control group (Figure S2F‐H). Overall, linc01503 could mediate GC tumorigenesis both in vitro and in vivo.

### 3.4 Linc01503 regulated cell cycle and apoptotic activities in GC

**FIGURE 5:**
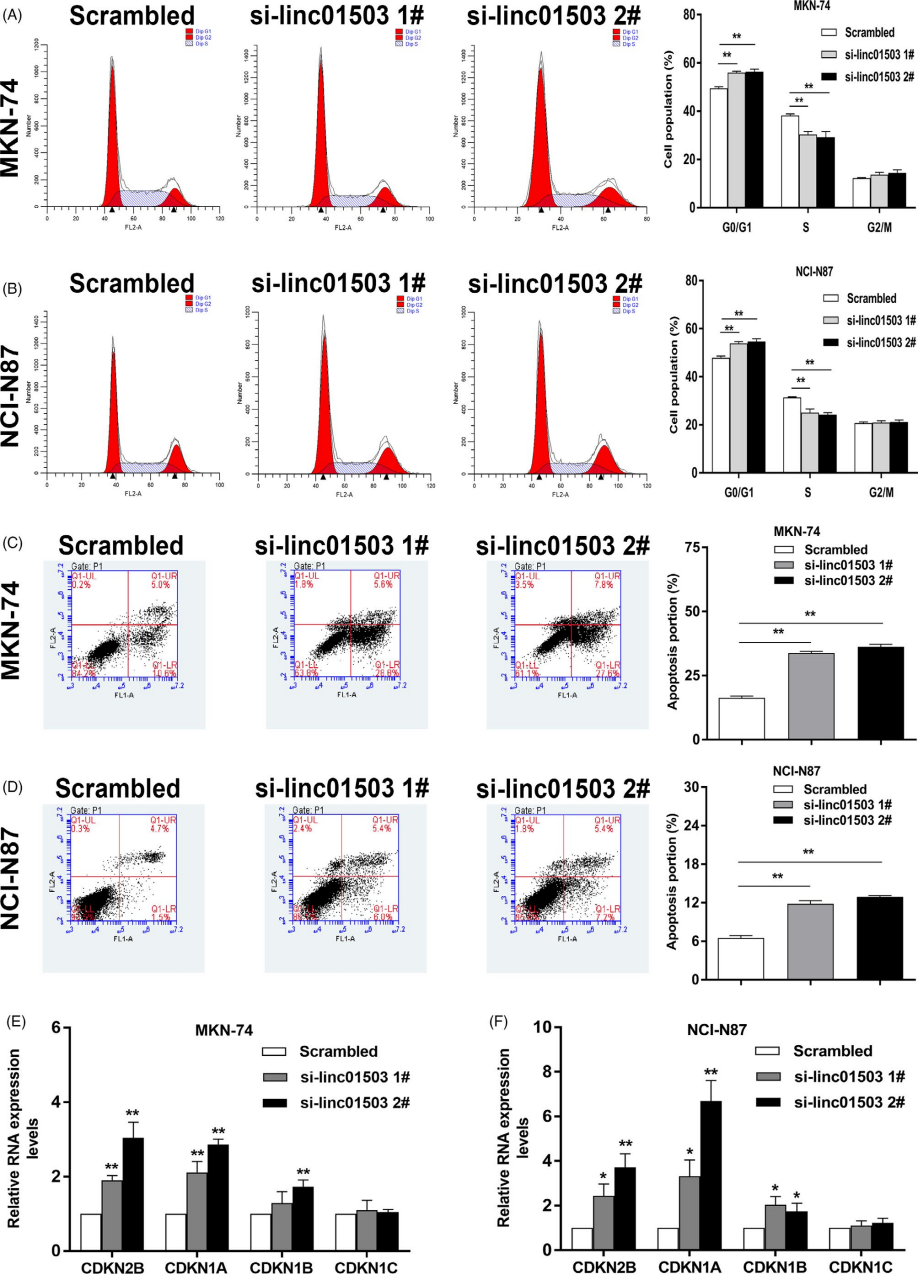
lncRNA linc01503 mediated GC cell cycle progression and apoptotic activities. A and B, The impact of linc01503 knockdown on cell cycle regulation in GC. C and D, The impact of linc01503 knockdown on GC cell apoptosis. E and F, The depletion of linc01503 effectively mediated the key genes associated with cell cycle and apoptosis in GC

The regulation of cancer cell cycle and apoptosis has been characterized to be tightly associated with cancer cell proliferation.^24^ To detect the impact of linc01503 on GC cell cycle regulation and apoptosis, we conducted flow cytometry assays in GC cells. Notably, an obvious increase at G1/G0 phase was observed in GC cells with linc01503 knockdown as compared with control group (Figure 5A, B). Additionally, silencing of linc01503 could markedly activate the apoptotic activities, leading to a significant upregulation of apoptotic rates in GC cells (Figure 5C, D). By contrast, overexpression of linc01503 obviously suppressed GC cell apoptosis (Figure S1C, D). Interestingly, we also detected the regulatory effects of linc01503 silencing on the key mediators closely associated with cell cycle and apoptosis, such as CDKN2B, CDKN1A and CDKN1B. As shown in Figure 5E, F, knockdown of linc01503 could significantly elevate the expression level of CDKN2B, CDKN1A and CDKN1B in both MKN74 and NCI‐N87 cells, further illuminating the crucial involvement of linc01503 in the regulation of GC cell cycle and apoptosis (Figure 5E, F).

### 3.5 Linc01503 served as a scaffold interacting with EZH2 and LSD1 in GC cells

**FIGURE 6:**
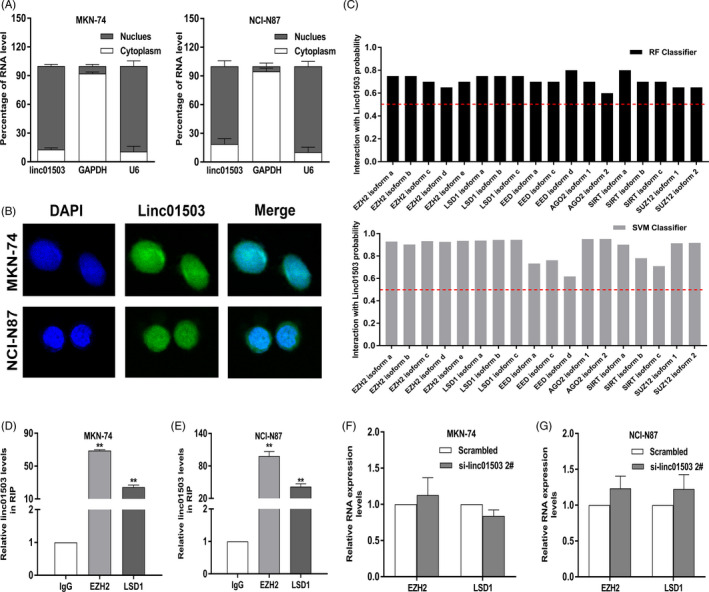
Linc01503 served as a scaffold interacting with EZH2 and LSD1 in GC cells. A and B, The subcellular localization and FISH analysis of the location of linc01503 in the cytoplasm and nuclear fractions in GC cells. C, Bioinformatics analysis of RNA‐binding proteins possibly bound with linc01503. D and E, Linc01503 could interact with EZH2 and LSD1 in both MKN‐74 and NCI‐N87 cells. F and G, The expression level of EZH2 and LSD1 kept unaltered in GC cells with linc01503 knockdown

To detect the regulatory mechanism of linc01503 in GC, we performed subcellular separation and FISH assays to clarify the distribution of lncRNA linc01503 in both MKN‐74 and NCI‐N87 cells. As depicted in Figure 6A, B, linc01503 was primarily distributed in the nucleus, implying the crucial involvement of linc01503 at the transcriptional level (Figure 6A, B). Interestingly, compelling evidence has highlighted that lncRNAs could interact with RNA‐binding proteins (RBPs) to induce gene expression regulation at transcription level.^25^, ^26^ Thus, we conducted bioinformatics analysis to predict the RBPs potentially bound with linc01503, such as EZH2, LSD1, EED, SIRT and SUZ12^27^ (Figure 6C).

Next, the exact associations between linc01503 and candidate RBPs were verified through RIP and qRT‐PCR assays. RIP assays found the presence of linc01503 in EZH2 and LSD1 immunoprecipitates from MKN74 and NCI‐N87 cell lysates (Figure 6D, E). Meanwhile, we examined the expression level of both EZH2 and LSD1 in linc01503‐depleted GC cells. Notably, both EZH2 and LSD1 level kept unaltered in GC cells with linc01503 knockdown, highlighting a specific association between linc01503 and EZH2/LSD1 in GC cells (Figure 6F, G). Together, linc01503 could act as a scaffold interacting with both EZH2 and LSD1 in GC cells.

### 3.6 DUSP5 and CDKN1A are key targets of linc01503 in GC cells

**FIGURE 7:**
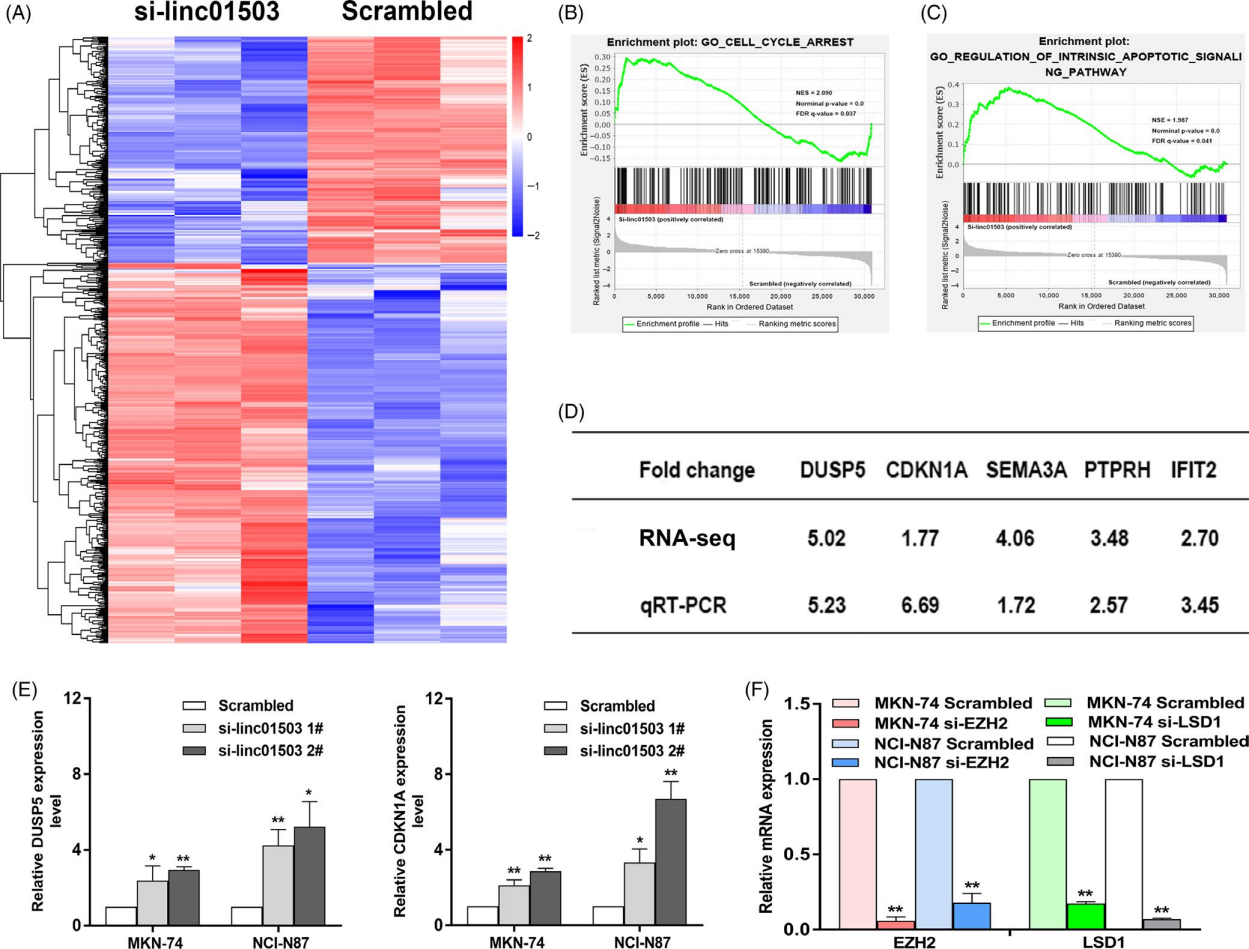
DUSP5 and CDKN1A are key downstream targets of linc01503 in GC. A, Heat map of affected candidate genes in GC cells from linc01503 knockdown group and control group. B and C, GSEA revealed the genes enriched by the depletion of linc01503 in GC. D, The expression levels of candidate genes induced by linc01503 knockdown were detected in GC cells. E, DUSP5 and CDKN1A were markedly elevated in GC cell with linc01503 knockdown. F, The knockdown efficacy of EZH2 and LSD1 was detected in both MKN‐74 and NCI‐N87 cells

To further identify the key downstream targets involved in linc01503‐mediated tumour‐promoting activities in GC, RNA‐seq analysis was performed in GC cells from control or linc01503 knockdown group (Figure 7A). GSEA analysis demonstrated that the gene signatures of cell cycle arrest and intrinsic apoptotic signalling pathway were much involved in linc01503‐depleted GC cells relative to control group (Figure 7B, C). Then, the expression levels of multiple candidate genes associated with the regulation of cell cycle and apoptosis were validated in GC cells, such as DUSP5, CDKN1A, semaphorin‐3A precursor (SEMA3A), protein tyrosine phosphatase receptor type H (PTPRH) and interferon‐induced protein with tetratricopeptide repeats 2 (IFIT2) (Figure 7D). Compelling evidence has demonstrated that DUSP5 and CDKN1A are key tumour suppressors in various cancers, mediating cell cycle and malignant progress.^28^, ^29^ Moreover, SEMA3A, PTPRH and IFIT2 were found to have significant effects on tumour inhibition and functioned as promising therapeutic agents for cancers.^30^, ^31^, ^32^ Notably, DUSP5 and CDKN1A were of particular interest owing to their remarkable upregulation in linc01503‐depleted GC cells relative to the control group (Figure 7D, E). Additionally, the expression level of EGR1 was also detected in GC cells with linc01503 knockdown through qRT‐PCR assays. It was found that EGR1 level kept unaltered in both MKN‐74 and NCI‐N87 cells with linc01503 knockdown compared with control group (Figure S3A, B). The mechanism behind this phenomenon requires further exploration in the future research.

**FIGURE 8:**
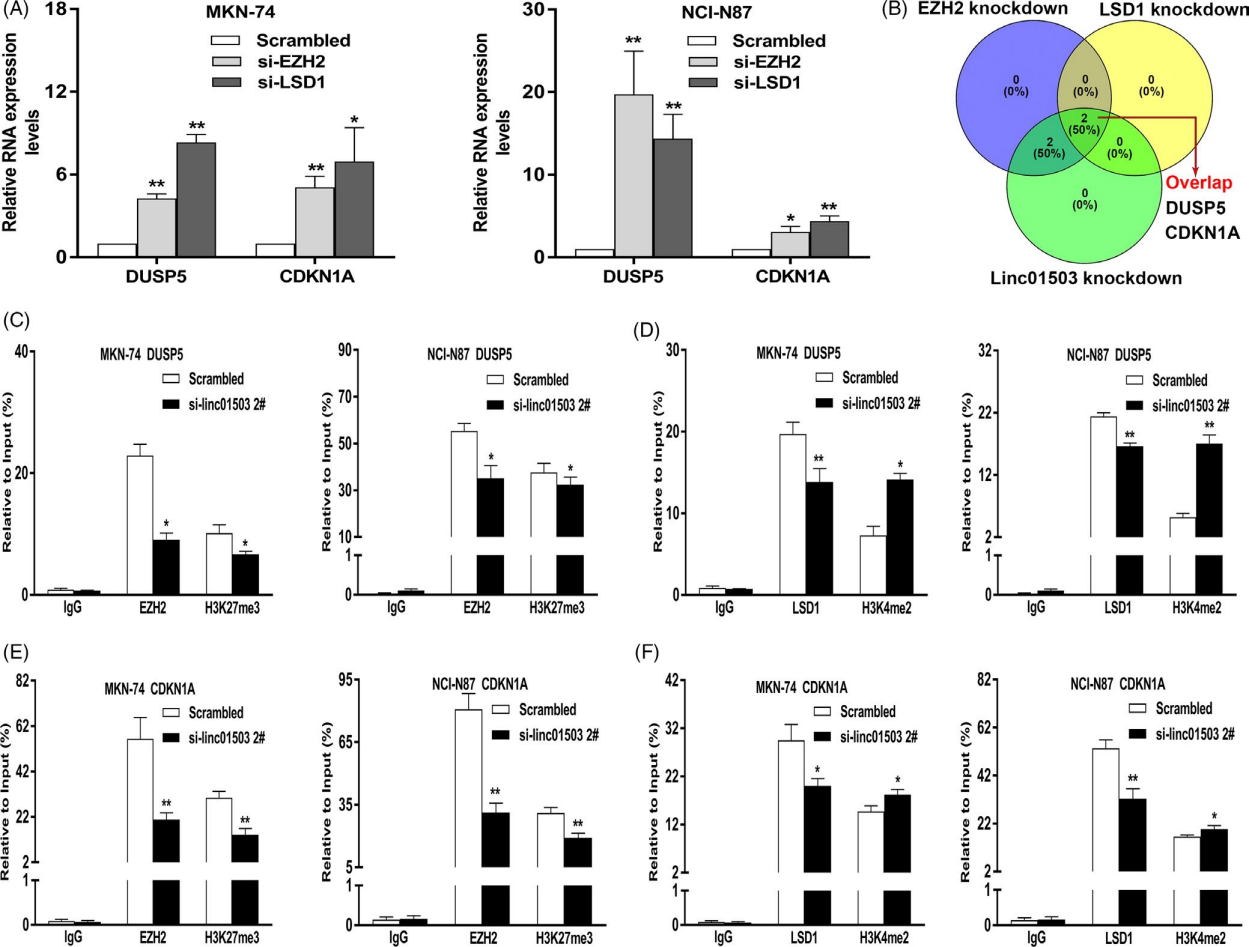
lncRNA linc01503 epigenetically silenced DUSP5 and CDKN1A expression by recruiting histone modification enzyme EZH2 and LSD1 in GC. A, DUSP5 and CDKN1A were obviously increased in GC cells with EZH2 or LSD1 knockdown. B, The Venn analysis highlighted the key targets co‐regulated by the depletion of linc01503, EZH2 and LSD1 in GC cells. C, ChIP‐qPCR assay of EZH2 and H3K27me3 occupancy in the DUSP5 promoter in GC cells transfected with linc01503 siRNA or Scrambled. D, ChIP‐qPCR assay of LSD1 and H3K4me2 occupancy in the DUSP5 promoter in GC cells transfected with linc01503 siRNA or Scrambled. E, ChIP‐qPCR assay of EZH2 and H3K27me3 occupancy in the CDKN1A promoter in GC cells transfected with linc01503 siRNA or Scrambled. F, ChIP‐qPCR assay of LSD1 and H3K4me2 occupancy in the CDKN1A promoter in GC cells transfected with linc01503 siRNA or Scrambled

Based on the current findings, it was found that linc01503 acted as a scaffold interacting with EZH2 and LSD1 in GC cells (Figure 6D, E). Thus, we further made assumptions that the candidate genes regulated by linc01503 knockdown may be possibly induced by the depletion of EZH2 or LSD1 in GC cells. To verify this hypothesis, the effective knockdown of EZH2 or LSD1 was made in both MKN‐74 and NCI‐N87 cells using siRNAs against EZH2 or LSD1 (Figure 7F). Intriguingly, both DUSP5 and CDKN1A were significantly increased in GC cells with knockdown of EZH2 or LSD1 (Figure 8A). The high overlap among linc01503, EZH2 and LSD1‐mediated regulation forcefully demonstrated the key regulatory axis of linc01503/EZH2/LSD1/overlapped targets.

### 3.7 LncRNA linc01503 mediated cell cycle progression and oncogenic activities through epigenetically silencing DUSP5 and CDKN1A expression in GC

As shown in Figure 8B, the Venn analysis showed that DUSP5 and CDKN1A were consistently up‐regulated and co‐regulated by linc01503, EZH2 and LSD1, highlighting that DUSP5 and CDKN1A are key targets of linc01503 in GC (Figure 8B). EZH2 and LSD1, which are enriched in various cancers, have been well‐recognized for their involvement in the epigenetic regulation.^33^, ^34^ Results shown in Figure 6D, E have found that linc01503 acted as a scaffold interacting with EZH2 and LSD1 in GC cells (Figure 6D, E). Thus, we made assumptions that linc01503 could impact epigenetic regulation on DUSP5 and CDKN1A through its association with EZH2 and LSD1 in GC cells.

To clarify the mechanism by which linc01503 induced the expression of DUSP5 and CDKN1A, the ChIP assays were performed in linc01503‐depleted GC cells and control cells. The results elucidated that EZH2 bound the promoter regions of DUSP5 and CDKN1A, and induced histone 3 lysine 27 trimethylation (H3K27me3) (Figure 8C, E). Meanwhile, the knockdown of linc01503 could markedly decrease these binding activities and H3K27me3 modifications (Figure 8C, E). As shown in Figure 8D, F, ChIP assays uncovered that the enrichment of LSD1 and H3K4me2 in the promoter region of DUSP5 and CDKN1A, which was remarkably reduced for LSD1 but markedly increased for H3K4me2 after transfections with si‐linc01503 (Figure 8D, F). Conclusively, lncRNA linc01503, presumably in part, could recruit EZH2/LSD1 and epigenetically repress DUSP5 and CDKN1A expression, thus contributing to oncogenic activities in GC.

## 4 DISCUSSION

Massive studies have elucidated that lncRNAs play important roles in the pathogenesis and progression of multiple cancers.^10^, ^21^, ^23^, ^26^ Currently, lncRNAs show great potential as targets and combined targeting of dysregulated lncRNAs may become a promising option for future cancer therapeutics.^35^, ^36^ Therefore, it is of great significance to enlarge the identification of lncRNAs and their underlying mechanisms, thereby facilitating the progress of lncRNA‐based treatments.

In the present study, publicly available data from GEO and TCGA data sets were downloaded and analysed to select candidate genes potentially associated with GC progression. Notably, linc01503 was markedly increased in GC and its elevation closely associated with poor survival for patients with GC. Interestingly, some of key transcription factors are highlighted to be involved in the dysregulation of lncRNAs.^23^ Based on the bioinformatics analysis, we uncovered that EGR1 was remarkably elevated in GC and its elevation elicited a positive association with linc01503 level in GC tissue samples, highlighting the crucial regulatory mechanism between EGR1 and linc01503 in GC. Moreover, silencing of EGR1 could significantly increase the apoptotic proportions in GC. Strikingly, we provided the first evidence of the upstream mechanism of linc01503 in GC and elucidated that the key transcription factor EGR1 critically activated the transcription of linc01503 in GC. Meanwhile, EGR1 level was found to keep unaltered in GC cells with knockdown compared with control group. It is an interesting topic to uncover the mechanism behind this phenomenon in the future research.

Functionally, silencing of linc01503 dramatically suppressed GC cell proliferation and activated the apoptotic activities, whereas overexpression of linc01503 elicited the opposite impact. Furthermore, knockdown of linc01503 resulted in a significant elevation of G1/G0 phase population in GC cells compared with the respective control groups. Subcellular separation and FISH assays showed that linc01503 was prevalently distributed in the nucleus, implying that linc01503 may be involved in the gene regulation at the transcriptional level. Importantly, bioinformatics analysis and RIP assays confirmed that linc01503 could serve as a scaffold interacting with EZH2 and LSD1 in GC cells. RNA‐seq assays combined with GSEA demonstrated that linc01503 knockdown dramatically affected genes associated with cell cycle arrest and apoptosis, potentially contributing to the promotion of GC proliferation. Interestingly, these potential targets were identified to be co‐regulated by the alteration of both EZH2 and LSD1. It was noted that DUSP5 and CDKN1A were consistently up‐regulated and co‐regulated by linc01503, EZH2 and LSD1, highlighting that DUSP5 and CDKN1A are key targets of linc01503 in GC. Finally, ChIP assays uncovered that linc01503 mediated transcriptional repression of DUSP5 and CDKN1A through H3K27 trimethylation and H3K4 demethylation in GC cells. Above all, lncRNA linc01503, at least partially, interacted with EZH2 and LSD1 to epigenetically silence the expression level of DUSP5 and CDKN1A in GC.

## 5 CONCLUSIONS

The regulatory axis of EGR1/linc01503/DUSP5/CDKN1A may be an attractive target for GC therapeutics. Our research may enrich the understanding of GC biology and accelerated the progress of lncRNA‐based therapeutics in GC or other types of cancers.

## CONFLICT OF INTEREST

The authors declare no competing financial interest.

## AUTHOR CONTRIBUTIONS

ZHM conceived the study, performed the experiments and drafted the manuscript. ZHM, XYG and YS participated in the figure drawing and data analysis. YS and XLW helped to conduct bioinformatics analysis. YY, XFX and JFJ provided full guidance throughout the entire research. All authors approved the final manuscript.

## Supporting information

Fig S1

Fig S2

Fig S3

Table S1

Appendix S1

## Data Availability

The data that support the findings of this study are available from the corresponding author upon reasonable request.
