# Supplementary figures and images for: EGR1‐mediated linc01503 promotes cell cycle progression and tumorigenesis in gastric cancer
Source: Cell Prolif. 2020 Nov 3;54(1):e12922. doi: 10.1111/cpr.12922 (PMC7791171; doi:10.1111/cpr.12922)

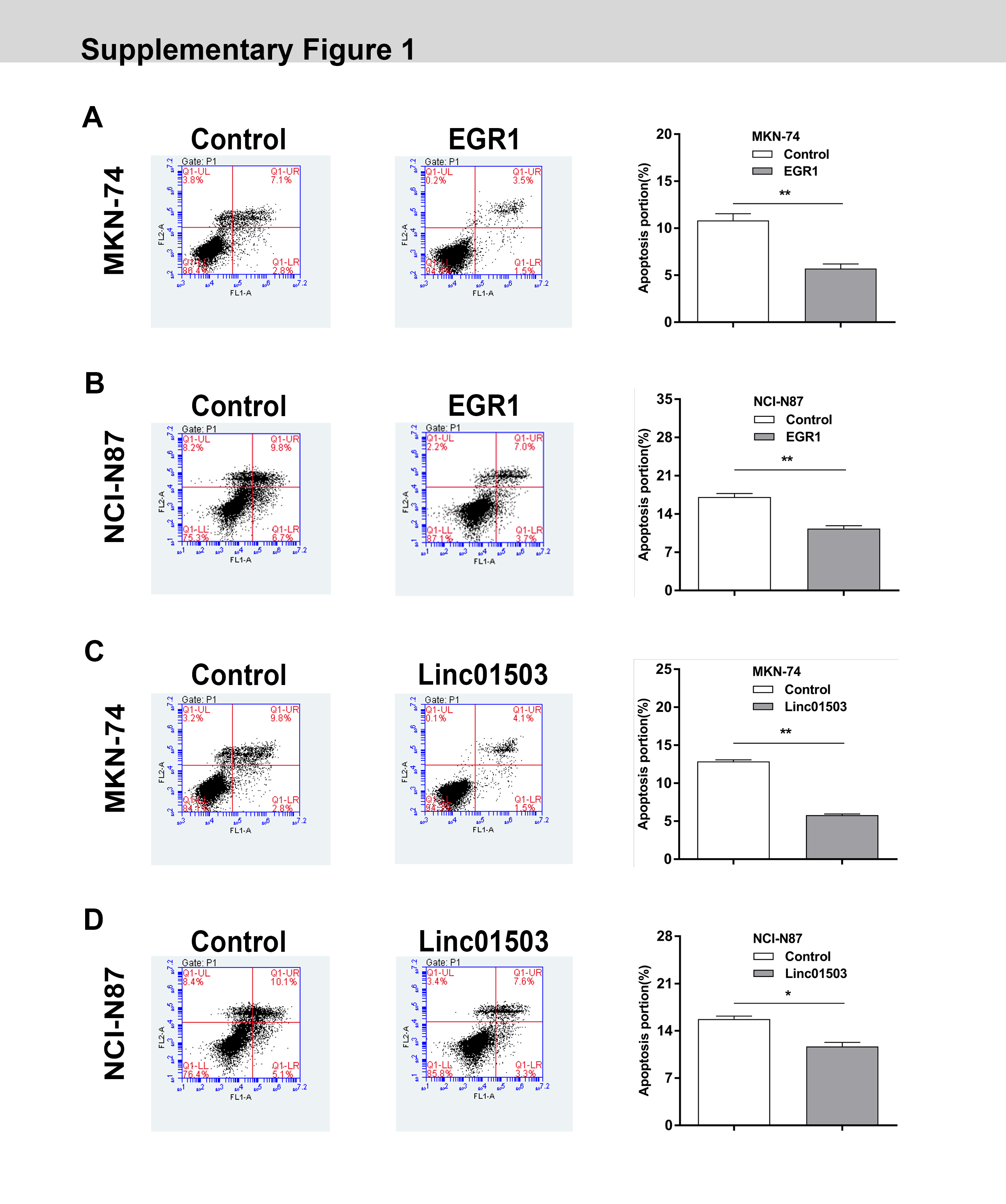

Supplement: Supplementary file 1 — Fig S1 [file CPR-54-e12922-s001.tif]

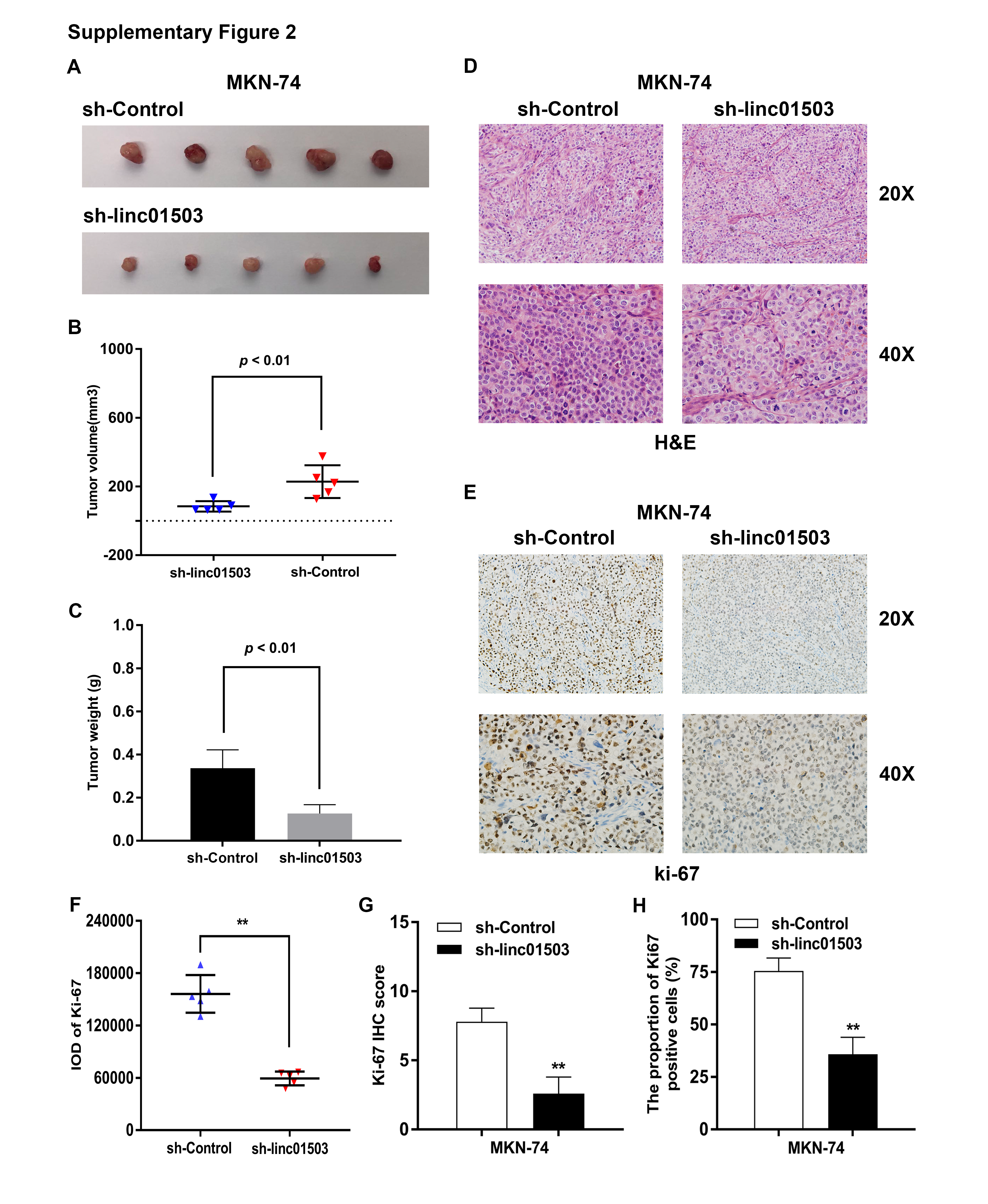

Supplement: Supplementary file 2 — Fig S2 [file CPR-54-e12922-s002.tif]

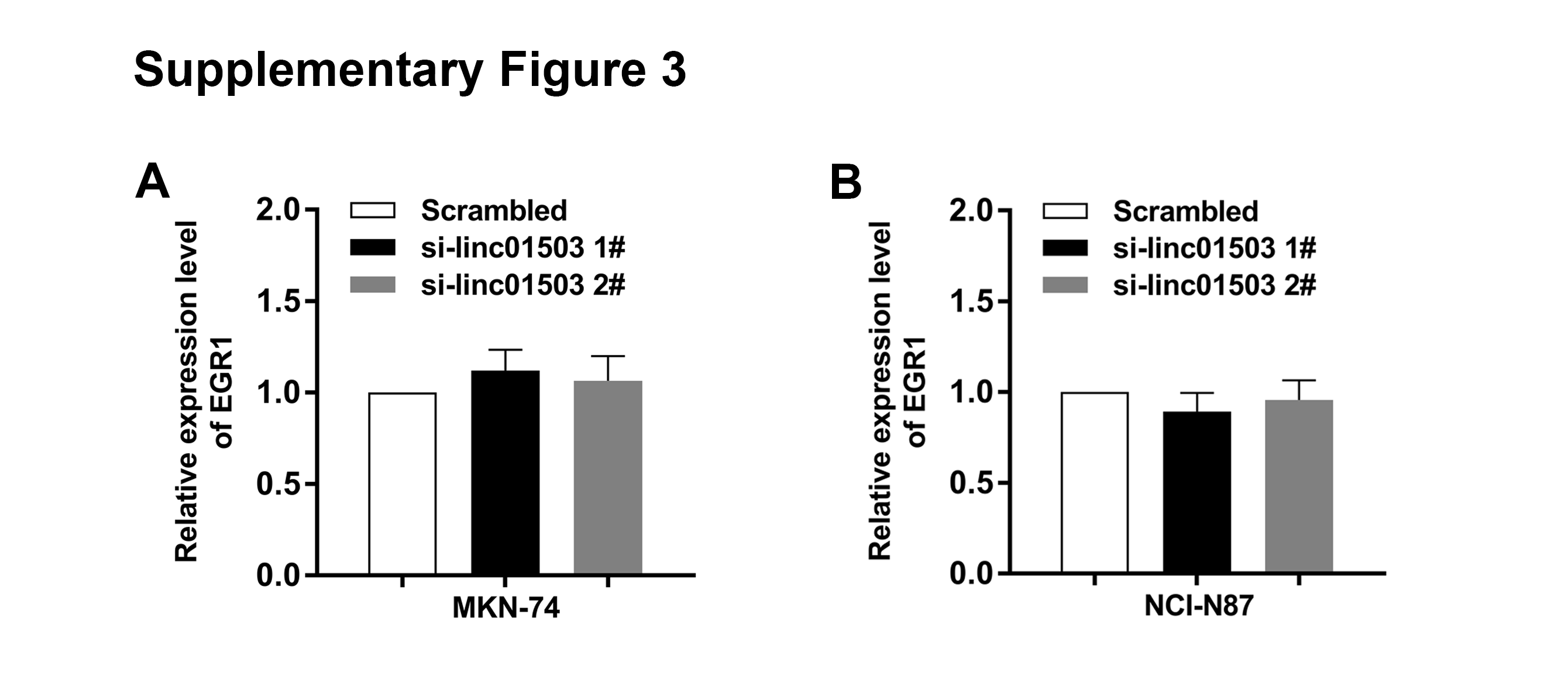

Supplement: Supplementary file 3 — Fig S3 [file CPR-54-e12922-s003.tif]
