# Supplementary material for: EGR1‐mediated linc01503 promotes cell cycle progression and tumorigenesis in gastric cancer
Source: Cell Prolif. 2020 Nov 3;54(1):e12922. doi: 10.1111/cpr.12922 (PMC7791171; doi:10.1111/cpr.12922)
Supplement: Supplementary file 4 — Table S1 [file CPR-54-e12922-s004.doc]

| **Supplementary Table 1. Primers for qRT-PCR, siRNAs oligonucleotides and the company for antibody.** | |
| --- | --- |
| **Primers used for qRT-PCR** | |
| Linc01503-F | TGGTCATCTTTGGGTGGAGC |
| Linc01503-R | TGACCCAGTCTCCTGTCAGT |
| GAPDH-F | GCTCTCTGCTCCTCCTGTTC |
| GAPDH-R | ACGACCAAATCCGTTGACTC |
| DUSP5-F | TCCCTGACTTCTAGCCCTGT |
| DUSP5-R | TTTAGCAGGATGTGGCCGTT |
| CDKN2B-F | ACGGAGTCAACCGTTTCGGGAG |
| CDKN2B-R | GGTCGGGTGAGAGTGGCAGG |
| CDKN1A-F | CAGCAGAGGAAGACCATGTG |
| CDKN1A-R | GGCGTTTGGAGTGGTAGAAA |
| CDKN1B-F | GATAATCCCGCTCTGAATGC |
| CDKN1B-R | CTGTATTTGGAGGCACAGCA |
| CDKN1C-F | CACGATGGAGCGTCTTGTC |
| CDKN1C-R | CCTGCTGGAAGTCGTAATCC |
| SEMA3A-F | CCTATGAACAATCGCCCAAT |
| SEMA3A-R | AGTGGAAAGCTCCATTGCTG |
| PTPRH-F | GCCCTTCAGCTTTGTAAGGA |
| PTPRH-R | GGCCACGTTCTCGTAGATGA |
| IFIT2-F | AGAGCAGCCTACGGCAACTA |
| IFIT2-R | GATTTCTGCCTGGTCAGCAT |
| EZH2-F | AGGAGTTTGCTGCTGCTCTC |
| EZH2-R | CCGAGAATTTGCTTCAGAGG |
| LSD1-F | CGGGCGAAGGTAGAGTACAG |
| LSD1-R | CGTCTCCATACCCTCCAGAA |
| **siRNAs oligonucleotides** |  |
| Linc01503 1# | UCGGAAUACCCACCUUUCUGGUAAU |
| Linc01503 2# | UGACAAGUGUGUACCUACGUGUCAG |
| Linc01503 3# | CAUGACCGUGUGGAGAAAGUUCUUU |
| si-EZH2 | CGGCUUCCCAAUAACAGUATT |
| si-LSD1 | CAUUUGAGGCUACUCUCCATT |
| **Antibody** | **Company** |
| GAPDH | Cell Signaling Technology |
| IgG | Millipore |
| EZH2 | Millipore |
| LSD1 | Millipore |
| H3K27me3 | Abcam |
| H3K4me2 | Abcam |
