# Supplementary material for: EGR1‐mediated linc01503 promotes cell cycle progression and tumorigenesis in gastric cancer
Source: Cell Prolif. 2020 Nov 3;54(1):e12922. doi: 10.1111/cpr.12922 (PMC7791171; doi:10.1111/cpr.12922)
Supplement: Supplementary file 5 — Appendix S1 [file CPR-54-e12922-s005.doc]

**Supplementary methods**

**Cell lines**

GC cell lines (MKN-74, NCI-N87, SGC-7901) and normal gastric epithelial cell line GES-1 were used in this study. NCI-N87, SGC-7901 and GES-1 cell lines were obtained from Shanghai Cell Research Institute, Shanghai, China. MKN-74 was obtained from JCRB (Japanese Collection of Research Bioresources Cell Bank, Japan). Cells were cultured in RPMI DMEM/1640 (Invitrogen, Shanghai, China) with 10% foetal bovine serum (FBS) at 37 °C in 5% CO2 and grown for no more than 20 passages in total for any experiment. Cell authentication was verified by short tandem repeat profiling and mycoplasma PCR testing of these cells was performed every month.

**RNA extraction and qRT-PCR assays**

The RNA concentrations were tested through 260/280 nm absorbance with a Nano-drop spectrophotometer (ND-100, Thermo Fisher Scientific, Massachusetts, USA). cDNA was synthesized from the isolated RNA using a Reverse Transcriptase kit (Takara, Dalian, China). The assays of qPCR were performed using SYBR Green (Takara). Glyceraldehyde 3-phosphate dehydrogenase (GAPDH) was used as endogenous control and the relative expression level of each gene was normalized to GAPDH.

**Cell transfection**

The linc01503 sequence was synthesized according to the full-length cDNA of human linc01503 and cloned into the expression vector pCDNA3.1 (Invitrogen). Knockdown of linc01503 were performed by lenti-virus-mediated shRNAs targeting linc01503 in GC cells. Moreover, three linc01503-targeting small interfering RNAs (siRNAs) were obtained from Invitrogen. Transfections were performed using Lipofectamine 3000 (Invitrogen) based on the instructions of the manufacturer.

**Animal models**

Four-week-old Balb/C nude mice were kept under specific pathogen-free (SPF) conditions in the Laboratory Animal Center of the Peking University Cancer Hospital. The animal programme was approved by the Animal Ethics Committee at Peking University Cancer Hospital. The tumors were generated by subcutaneously injecting GC cells from linc01503 stable knockdown or control group. Tumour volume was calculated using the following equation: V=0.5*D*d2 (V, volume; D, longitudinal diameter; d, latitude diameter). The mice were sacrificed and tumors were stripped for further analysis (weight measurement and IHC).

**RNA immunoprecipitation (RIP)**

The GC cells were harvested in ice-cold PBS and lysed with RIP lysis buffer. Then, specific antibodies and negative control IgG were implicated for the immunoprecipitations. The co-incubations of RIP lysates, antibodies and magnetic beads were performed at 4°C for 6 hours. Thereafter, the beads were washed with RIP wash buffer and incubated with proteinase K for 30 minutes at 55 °C. After removal of proteins of the beads, the purified RNA was eluted and analyzed for the presence of linc01503 through qRT-PCR assays.

**Chromatin immunoprecipitation assays (ChIP)**

The cells were firstly seeded in the six-well plates and treated with formaldehyde (1%) and glycine (125 nM), respectively. The cell lysates were sonicated to produce DNA fragments, which were thereafter immunoprecipitated with specific antibodies or IgG antibody, respectively. Finally, the ChIP-derived DNA was assessed by qRT-PCR assays.

**Subcellular separation and fluorescence in situ hybridization (FISH) assays**

Subcellular separation assays were performed with a PARIS Kit (Life Technologies) in accordance with the protocol of the manufacturer. The cytoplasmic and nuclear RNA were assessed through qRT-PCR assays. GAPDH and U6 were used as cytoplasmic and nuclear controls, respectively. FISH assays were performed with a Ribo™ Fluorescent In Situ Hybridization Kit (RiboBio, Guangzhou, China) according to the manufacturer’s instructions. The images were captured using LSM 800 confocal microscope (Carl Zeiss, Jena, Germany). Nuclei was labeled with DAPI (blue) and linc01503 was labeled by the RNA probe-linc01503 (green).

**Cell proliferation assay**

The treated cells were seeded and cultured in a 96-well plate at 37 °C in 5% CO2. Then,, 10 ul Cell Counting Kit-8 (CCK8; Beyotime Institute of Biotechnology, Shanghai, China) solution was added into each well and let incubation for another 2 hours at 37 °C in 5% CO2. The optical density 450 nm (OD450) was measured using a microplate reader. For colony-formation assays, the treated cells were seeded in 6-well plates to be cultured for 7 days. Then, the cells were fixed by methanol for 15 minutes and stained by 0.1% crystal violet solution (548-62-9, Aladdin, China) at room temperature for 30 min, and washed by PBS twice.

**Flow cytometry assay**

For cell cycle analysis, the treated cells were fixed using 70% ethanol overnight at −20 °C. Then, the harvested cells were stained with PI using a CycleTEST PLUS DNA kit (BD Biosciences). In total, 10,000 cells were acquired on C6 Flow Cytometer and FACScan analysis was performed to calculate the percentage of cells in the phase of G0/G1, S or G2/M. As for apoptosis assays, a FITC-Annexin V cell apoptosis assay kit (BD Biosciences) was used to stain the harvested cells with FITC/PI. After 15 minutes in dark, cells were acquired and analyzed through C6 Flow Cytometer to detect the apoptotic rates.

**Immunohistochemical (IHC) analysis**

The tissue samples were fixed overnight in a formalin solution, dehydrated in ethanol, embedded in paraffin. Then, it was sectioned and stained with haematoxylin and eosin (HE). Immunohistochemistry for Ki67 was also performed on paraffin slides of mouse xenograft tumors using anti-Ki67. For measurement IOD, five discontinuous visual fields were selected under a 400X light microscop. Then, the Image J software was implicated to measure the IOD of ki-67 in each image. The staining intensity was scored as 0 (negative), 1 (weak), 2 (moderate) and 3 (strong), and cell staining was classified according to the the percentage of positive cells: 0 (0% to 5%), 1 (6% to 25%), 2 (26% to 50%), 3 (51% to 75%), 4 (76% to 100%). The ki-67 IHC score was calculated by multiplying the number corresponding to different ki-67 staining intensity and staining regions.
